# Genetic Polymorphisms of *IFNG* and *IFNGR1* with Latent Tuberculosis Infection

**DOI:** 10.1155/2019/8410290

**Published:** 2019-10-10

**Authors:** Shouquan Wu, Xiangmin Liu, Yu Wang, Miaomiao Zhang, Minggui Wang, Jian-Qing He

**Affiliations:** Department of Respiratory and Critical Care Medicine, West China Hospital, Sichuan University, Chengdu, Sichuan, China

## Abstract

Previous studies indicated that single-nucleotide polymorphisms (SNPs) of interferon gamma (IFNG) and IFNG receptor 1 (IFNGR1) may be involved in the pathogenesis of pulmonary tuberculosis (PTB) in different populations. In order to further explore the results in a Chinese Han population, this study was designed to investigate potential associations between the polymorphisms in *IFNG* and *IFNGR1* and susceptibility to latent tuberculosis infection (LTBI) and/or PTB in a Chinese Han population. A total of 209 PTB, 173 LTBI, and 183 healthy control subjects (HCS) were enrolled in our study. Genotyping was conducted using an improved multiplex ligase detection reaction (iMLDR). We performed a logistic regression including sex and age as covariates to test the effect of alleles/genotypes on LTBI and/or TB. All six markers studied in *IFNG* and *IFNGR1* conformed to the Hardy–Weinberg equilibrium (HWE). The *IFNG* rs1861494 was significantly associated with LTBI in recessive model, and the CC+CT genotype decreased risk of LTBI by 50% (*P* = 0.046, OR = 0.50, 95%CI: 0.25-0.99). The *IFNGR1* rs2234711 was significantly associated with LTBI, and allele A increased the risk of LTBI by 55% (*P* = 0.047, OR = 1.55, 95%CI: 1.00-2.40). In the present study, we found that *IFNG* and *IFNGR1* polymorphisms were associated with LTBI.

## 1. Introduction

Tuberculosis (TB) is an ancient disease and severely affects human health all over the world. In 2017, there were approximately 889,000 new cases of TB, and 38,800 people died from this disease in China [1]. *Mycobacterium tuberculosis* (*M.TB*) infects almost 33% of the world's population. However, only 5-10% of them develop active TB disease with clinical symptoms. The differences in the infection outcome are still unknown.

It was suggested that host genetic factors and *M.TB* itself may influence the outcome of *M.TB* infection [2]. Anthropological studies based on the polymorphic gene suggested that susceptibility to infectious diseases was related to genetic diversities of polymorphic genes [3]. Moreover, host genetic variants play a critical role in the TB progression in humans [4, 5]. It was demonstrated that the determination of the heritability to develop TB varies from 39% to 71% [6, 7]. Other factors including innate and adaptive immunity are also thought to affect the progression of TB. Previous studies have revealed that several immune-related genes, including IL-1B [4], IL-6 [4], IL-8 [8], IL-12B [9], and TNF [4], may contribute to *M.TB* infection. Therefore, the major elements of host genetic susceptibility to TB may be based on the population-based variants in adaptive and innate immunity [10]. TB development is regulated by different immunocytes and relies on the interaction of cytokines secreted by these cells [11].

Interferon gamma (IFNG) was chosen in this study because of its critical role in the immune system at multiple levels [12]. *IFNG* is located in chromosome 12q15, and it has four exons. When *M.TB* invades the host, IFNG activates macrophages, which act as the first line of host defense against this pathogen and kills it [13]. Individuals who suffer from inherited disorders of IFNG-mediated immunity are more likely to be infected with *M.TB* [14]. Evidence suggested that disturbance of IFNG production resulted in varied clinical presentations [15]. What is more, polymorphisms in *IFNG* were variably associated with TB among different populations [16].

IFNG receptor 1 (*IFNGR1*) gene is located in chromosome 10 and encodes one chain of the IFNGR1. IFNGR1 is a pleiotropic cytokine secreted by NK and T cells [14]. IFNG regulates gene expression by interacting with IFNGR1 through its homodimers [17, 18]. Mice with disrupted *IFNGR1* are more likely to be infected with *M.TB* [19]. It has been demonstrated that IFNGR1 genotype and clinical phenotype were associated [20].

Most previous studies have evaluated the association between TB and *IFNG*/*IFNGR1* polymorphisms but have not differentiated those with LTBI and those without TB infection in the control group. In this study, we aim to investigate the association of *IFNG*/*IFNGR1* polymorphisms with healthy control subjects (HCS), latent tuberculosis infection (LTBI), and pulmonary TB (PTB).

## 2. Materials and Methods

### 2.1. Subjects

A total of 209 PTB and 356 close contacts of individuals with sputum-positive PTB were enrolled between 2013 and 2014. The selection of sample size was based on previous studies [21–23]. All of the participants were recruited from the West China Hospital of Sichuan University (Sichuan, China), and they are all genetically unrelated Chinese Han people. All cases and controls (matched for sex) were over 16 years old. PTB cases were all bacteriologically confirmed patients who were diagnosed by sputum culture, and/or percutaneous needle aspiration biopsy of the lung, and/or lung lavage by bronchoscopy. We differentiated close contacts of PTB to LTBI subjects and HCS depending on interferon gamma release assay (IGRA) results, symptoms, chest X-ray, and sputum examination. The definition of close contacts is as follows: (1) shared airspace with PTB for at least 15 hours per week for at least one week during an infectious period and (2) shared airspace with a PTB patient for at least 180 hours during an infectious period [24]. LTBI and HCS had no TB-related symptoms and had negative sputum acid-fast bacilli smear for *M.TB*. None of the participants were reported to have complications of chronic obstructive pulmonary disease, HIV, hepatitis B and/or C, or immune-mediated disorders.

Two to five milliliters of venous blood specimen was drawn from each of the participants after they agreed to attend this research and signed an informed consent. The blood sample was collected by using ethylene diamine tetra acetic acid (EDTA) tubes and then stored in a -80°C freezer for further investigation. We further extracted the DNA from the blood using a genomic DNA purification kit (Axygen Scientific, Inc., Union City, CA, USA) in accordance with the manufacturer's instructions. The DNA specimens were stored at -80°C for further genotyping. This study was approved by the ethical committee of the West China Hospital Institutional Review Board.

### 2.2. SNP Selection and Genotyping

Tag-SNPs (SNPs in a genome region with high linkage disequilibrium that represents a set of SNPs) [25] of IFNG/IFNGR1 were chosen to detect the association between TB and IFNG/IFNGR1. We selected the tag-SNPs from the HapMap database (http://www.hapmap.org) based on the following criteria: (1) minor allele frequency (MAF) ≥ 0.1 in the Chinese Han population; (2) *P* value for the Hardy–Weinberg equilibrium (HWE) test ≥ 0.05; and (3) *r*^2^ of pairwise linkage disequilibrium (LD) ≤ 0.8. SNP genotyping was performed using the improved multiplex ligase detection reaction (iMLDR), with technical support from the Shanghai Genesky Biotechnology Company. To validate the genotype results, 5% of samples underwent repeat iMLDR.

### 2.3. IFNG Release Assay

A total of 59 PTB and 173 LTBI underwent IFNG release assay QuantiFERON-TB Gold In-Tube (QFT-GIT) according to the manufacturer (Cellestis, Carnegie, Australia). The QFT-GIT results were decided based on the cut-off values suggested by the manufacturer.

### 2.4. Statistical Analysis

Continuous variables were tested using the Student *t*-test. The *χ*^2^-test was used to analyze HWE by comparing the observed and expected genotype frequencies among participants. Logistic regression analysis adjusted for sex and age was used to calculate the differences in the allele frequencies and genotype distribution between the groups. We further conducted a haplotype analysis by constructing haplotypes utilizing the SHEsis online software (http://analysis.bio-x.cn). Power analysis was conducted by using the Power and Sample Size Calculation Software (http://biostat.mc.vanderbilt.edu/PowerSampleSize). *P* values smaller than 0.05 were considered significant. Data were calculated with SPSS software (SPSS, Inc., Chicago, IL, USA).

## 3. Results

### 3.1. Basic Information of Study Subjects

The general information of the three study groups is shown in Table 1. We recruited 209 PTB (107 males and 102 females), 173 LTBI (83 males and 90 females), and 183 HCS (84 males and 99 females) in this study. LTBI was the control when compared with PTB, and HCS was the control when compared with LTBI. Grouping of LTBI and HCS resulted from IGRA tests. The mean of age was 38.76 (±16.97) years for the PTB group, 50.34 (±15.92) years for the LTBI group, and 46.98 (±14.74) years for the HCS. There was no significant difference in sex distribution between groups. However, the distribution of age was significantly different in the three groups. We therefore performed the logistic regression including age as a covariate to test the effect of genotype on LTBI/TB.

### 3.2. Characteristics of SNPs

A total of six tag-SNPs (2 in *IFNG* and 4 in *IFNGR1*) were selected in the study. The basic information of all tag-SNPs including chromosome location, functional consequence, MAF, and *P* values for HWE is listed in Table 2. None of the SNPs were found to deviate from HWE in any group.

### 3.3. Association Analyses of *IFNG* and *IFNGR1* Polymorphisms with LTBI/HCS

As shown in Table 3, one SNP (rs1861494) in *IFNG* and one SNP (rs2234711) in *IFNGR1* are significantly associated with LTBI compared with HCS. The rs1861494 polymorphism was significantly associated with decreased risk for LTBI under the recessive model (*P* = 0.046, OR = 0.50, 95%CI: 0.25-0.99). The rs2234711 AA genotype was significantly associated with increased risk for LTBI in the dominant model (*P* = 0.047, OR = 1.55, 95%CI: 1.00-2.40). However, no significant association was found for PTB when comparing the LTBI group with the PTB group in any genetic model.

The haplotype association results are shown in Table 4. As for *IFNGR1*, the GATC haplotype was significantly associated with increased risk for LTBI (*P* = 0.033, OR = 1.39, 95%CI: 1.03-1.89). No haplotypes in *IFNGR1* exhibited a significant association with LTBI/PTB.

### 3.4. Association between *IFNG* and *IFNGR1* Polymorphisms and IGRA Results

**Figure 1:**
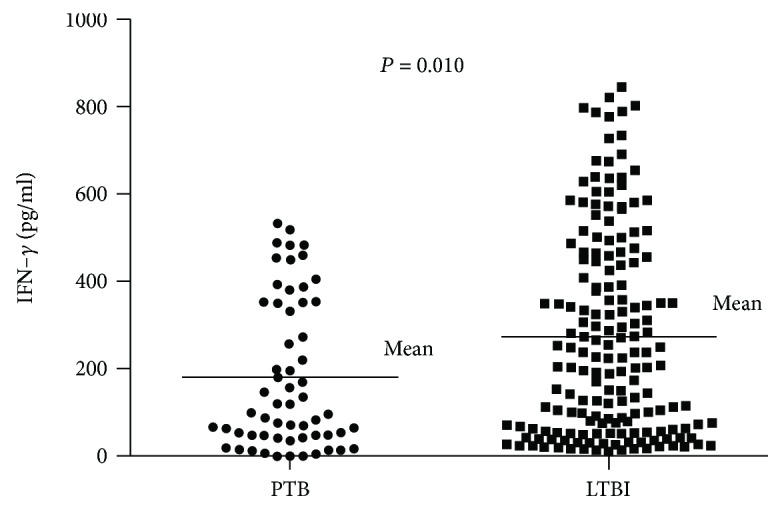
Evaluation of IFNG production levels among the LTBI and PTB.

We observed a positive IGRA result in 86.4% of the PTB. We then studied the IFNG production in IGRA (pg/ml) between PTB and LTB. As shown in Figure 1, the LTBI group had a higher level of IFNG production (275.56 pg/ml) than had the PTB group (181.50 pg/ml). Furthermore, we performed an analysis of the association between IGRA results (IGRA positive or IGRA negative) and IFNG/IFNGR1 polymorphisms. Three genetic models were used to test this relationship, and the results showed that polymorphisms in IFNG/IFNGR1 are not associated with IGRA results (Table 3).

### 3.5. Power Analysis

We used the odds ratio (OR) of 2.0, 3.0, and 4.0 to calculate the power of the sample size for each SNP. The result suggested that the sample size provided reasonable power (>80%) to draw conclusions with OR 2.0 or above (Table 5).

## 4. Discussion

Our study was designed to evaluate whether tag-SNPs in *IFNG*/*IFNGR1* could influence the susceptibility to LTBI/PTB and IGRA results. We performed the association study in three groups: PTB, LTBI, and HCS. We demonstrated that *IFNG* rs1861494 polymorphism was associated with decreased risk of LTBI. A significant association was also observed for *IFNGR1* rs2234711 polymorphism and LTBI. We also revealed that *IFNG*/*IFNGR1* polymorphisms did not affect IGRA results.

When the *M.TB* pathogen invades the host, the innate immune response will be activated, which includes the production of IFNG by natural killer (NK) and NK T cells. Once antigen-specific immunity develops, IFNG will be produced by CD4 and CD8 T cells [26]. It has been suggested that mice with disruption of *IFNG* compared with wild-type mice were more likely to be infected with *M.TB* [19]. Along with investigations with mice that demonstrated that *IFNG* plays a critical role in *M.TB* infection, some evidence in clinical studies has suggested the importance of IFNG in controlling *M.TB* infection in humans [14, 27]. High IFNG levels at the infected sites indicate that this cytokine may be an important determinant of TB [28]. Thus, this evidence reveals the critical role of IFNG in TB. Therefore, genetic polymorphisms in *IFNG* have been biologically plausible candidate markers associated with individual susceptibility.

The *IFNG* was the best-studied candidate gene in terms of its relationship with TB. However, results of previous studies were inconsistent. In the present study, two tag-SNPs (rs1861494 and rs2069718) of IFNG were genotyped. *IFNG* rs1861494, located in intron 3, have a differential affinity to bind putative nuclear factor [29]. It was suggested that rs1861494 can alter gene transcription, which might have a functional consequence on IFNG expression [30]. The rs1861494 has been associated with inflammatory bowel disease [31], IgA nephropathy [32], hepatitis B virus [33], and asthma [29]. Several studies have been conducted to investigate the association between rs1861494 polymorphism and tuberculosis, but the results were inconsistent. A study from Argentina showed that rs1861494 polymorphism was associated with tuberculosis resistance in a dominant model [34]. Also, another study demonstrated that GG genotype was related to tuberculosis [35]. Besides, two studies showed no association between this SNP and tuberculosis [22, 36]. However, in contrast to the aforementioned findings, we found that CC genotype was protected against LTBI in a recessive model. The rs2069718, located in intron 3, was reportedly associated with systemic lupus erythematosus [37] and tuberculosis [35]. However, this SNP showed no significant association with LTBI/TB in our study. The discrepancy may be due to the study design that differentiates LTBI subjects from subjects without TB infection.

IFNG pathway is mediated by the ligand binding to IFNGR1. It was reported that inherited IFNGR1 deficiency was found in several kindreds, indicating an association of mutations in the *IFNGR1* gene with susceptibility to weakly pathogenic mycobacteria [38]. The functional SNP rs2234711, located in the 5′-UTR region of the *IFNGR1* gene, encodes the human IFNGR ligand-binding chain I. Juliger et al. found that the switch from T to C at rs2234711 in the promoter region might reduce the expression level of IFNGR1 at the cell surface [39]. Several studies have been conducted to investigate the association between the rs2234711 polymorphism and tuberculosis susceptibility but have yielded inconsistent results. A previous study conducted in an African population suggested that the prevalence of tuberculosis was lower in African populations with the minor alleles of rs2234711, revealing a protective effect [17]. In a Chinese study, the protective effect of rs2234711 against tuberculosis has also been demonstrated [40]. However, the association between IFNGR1 and LTBI/tuberculosis has not been studied. Our results differ from those of the aforementioned literature in that we found a significant association between rs2234711 polymorphism and the development of LTBI from HCS in a Chinese population. It was reported that rs1327475 was associated with increased risk for the development of TB [40], while this result was not replicated in our study. Another two SNPs rs3799488 and rs9376267 were suggested to be not associated with tuberculosis [40]. Similarly, our study did not show a significant association between the risk of LTBI/PTB and these two SNPs. For the first time, our findings provide a clue for exploring the effects of IFNG/IFNGR1 SNPs on the prevalence of TB beyond the control of infection acquisition.

We investigated whether IFNG/IFNGR1 polymorphisms were associated with QFT-GIT results. Among the PTB subjects, the sensitivity of the QFT-GIT was 86.4%. The result was similar to those of previous studies [41, 42]. In our study, *IFNG*/*IFNGR1* polymorphisms were not associated with positive and negative QFT-GIT results. The result was the same in a study conducted in the Canadian population [41]. We also evaluated the IFNG production among LTBI and PTB participants, and the results suggested that the LTBI group has higher mean IFNG production than the PTB group. We speculated that at the stage of *M.TB* infection, the cell-mediated immune response was still reasonably strong to prevent progression to TB.

## 5. Conclusions

Functional polymorphisms in the *IFNG*/*IFNGR1* genes have shown significant associations with LTBI. The results we identified may help in further research on the potential role of the *IFNG*/*IFNGR1* pathway in human immune responses to *M.TB* infection and progression to active TB.

## Figures and Tables

**Table 1:** Baseline information on the study groups.

	PTB (*n* = 209)	LTBI (*n* = 173)	HCS (*n* = 183)	PTB vs. LTBI *P* value	LTBI vs. HCS *P* value
Age, mean ± SD	38.76 ± 16.97	50.34 ± 15.92	46.98 ± 14.74	<0.001	0.031
Male, *N* (%)	107 (0.51)	83 (0.48)	84 (0.46)	0.539	0.750
Symptoms and signs		None	None		
Cough	153				
Hemoptysis	31				
Dyspnea	51				
Night sweats	68				
Thoracalgia	31				
Fever	82				
Lung rale	37				

SD: standard error; PTB: pulmonary tuberculosis; LTBI: latent tuberculosis infection; HCS: healthy control subject.

**Table 2:** Basic information of studied SNPs.

Gene/SNPs	Chromosome	Location	Functional consequence	MA	MAF	MA	MAF	HWE	
				LTBI		HCS		LTBI	HC
*IFNG*									
rs1861494T>C	12	68551409	Intron 3	C	0.30	C	0.36	0.797	0.614
rs2069718A>G	12	68550162	Intron 3	G	0.17	G	0.13	0.320	0.914
*IFNGR1*									
rs1327475G>A	6	137536455	5′ flanking	C	0.10	C	0.13	0.291	0.103
rs2234711G>A	6	137540520	5′ UTR_exon1	G	0.41	G	0.34	0.961	0.651
rs3799488T>C	6	137519780	Intron 6	C	0.27	C	0.29	0.804	0.976
rs9376267C>T	6	137531031	Intron 1	C	0.46	C	0.43	0.801	0.860

Abbreviations: SNP: single-nucleotide polymorphism; LTBI: latent tuberculosis infection; HCS: healthy control subjects; MA: minor allele; MAF: minor allele frequency; HWE: Hardy–Weinberg equilibrium.

**Table 3:** Association of *IFNG* and *IFNGR1* polymorphisms with different groups.

Genes/SNPs	Genetic model	LTBI vs. PTB		HCS vs. LTBI		IGRA (+) vs. IGRA (-)^∗^	
		*P* ^#^	OR (95%CI)^#^	*P* ^#^	OR (95%CI)^#^	*P* ^#^	OR (95%CI)^#^
*IFNG*							
rs1861494	Allelic	0.440	1.14 (0.80-1.57)	0.166	0.80 (0.58-1.10)	0.428	0.61 (0.18-2.08)
T>C	Dominant	0.627	1.11 (0.72-1.72)	0.573	0.89 (0.58-1.35)	0.675	0.70 (0.13-3.76)
	Recessive	0.368	1.40 (0.68-2.89)	0.046	0.50 (0.25-0.99)	0.188	0.15 (0.01-2.50)

rs2069718							
A>G	Allelic	0.477	0.86 (0.56-1.32)	0.194	1.33 (0.87-2.03)	0.689	1.56 (0.18-13.64)
	Dominant	0.601	0.88 (0.54-1.43)	0.368	1.25 (0.77-2.01)	0.733	1.49 (0.15-14.87)
	Recessive	0.439	0.58 (0.15-2.30)	0.113	3.76 (0.73-19.28)	—	

*IFNGR1*							
rs1327475	Allelic	0.075	1.56 (0.96-2.54)	0.159	0.71 (0.44-1.14)	0.390	0.47 (0.09-2.62)
G>A	Dominant	0.070	1.63 (0.96-2.75)	0.131	0.68 (0.41-1.13)	0.360	0.40 (0.06-2.82)
	Recessive	—		—		—	

rs2234711							
G>A	Allelic	0.360	0.87 (0.64-1.18)	0.055	1.35 (0.99-1.83)	0.520	1.49 (0.45-4.95)
	Dominant	0.306	0.79 (0.50-1.24)	0.047	1.55 (1.00-2.40)	0.972	1.03 0.17-6.27)
	Recessive	0.694	0.89 (0.50-1.58)	0.335	1.33 (0.75-2.35)	—	

rs3799488							
T>C	Allelic	0.368	0.85 (0.60-1.21)	0.471	0.89 (0.64-1.23)	0.536	0.68 (0.20-2.30)
	Dominant	0.212	0.76 (0.49-1.17)	0.674	0.91 (0.60-1.39)	0.214	0.32 (0.05-1.93)
	Recessive	0.761	1.16 (0.44-3.03)	0.325	0.65 (0.27-1.54)	—	

rs9376267							
C>T	Allelic	0.987	0.99 (0.74-1.35)	0.175	0.81 (0.61-1.10)	0.884	0.92 (0.29-2.90)
	Dominant	0.437	1.21 (0.75-1.95)	0.157	0.71 (0.44-1.14)	0.538	0.49 (0.05-4.70)
	Recessive	0.374	0.79 (0.46-1.34)	0.443	0.83 (0.51-1.35)	0.683	1.61 (0.16-15.96)

SNPs: single-nucleotide polymorphisms; CI: confidence interval; OR: odds ratio; PTB: pulmonary tuberculosis; LTBI: latent tuberculosis infection; HCS: healthy control subjects; IGRA: interferon gamma release assay. ^#^Adjusted by age and sex status, ^∗^in pulmonary tuberculosis group, +/- positive/negative.

**Table 4:** Haplotypes of the *IFNG* and *IFNGR1* genes and their distributions in the three groups.

Gene/haplotype	LTBI vs. PTB				HCS vs. LTBI			
	Case (%) *n* = 418	Control (%) *n* = 344	*P*	OR (95%CI)	Case (%) *n* = 344	Control (%) *n* = 366	*P*	OR (95%CI)
*IFNG*								
CA	141.99 (0.34)	105.98 (0.31)	0.354	1.20 (0.85-1.57)	105.98 (0.31)	131.98 (0.36)	0.138	0.79 (0.58-1.08)
CG	0.01 (0.00)	0.02 (0.00)	—	—	0.02 (0.00)	0.02 (0.00)	—	—
TA	219.01 (0.52)	183.02 (0.53)	0.824	0.97 (0.73-1.29)	183.02 (0.53)	186.02 (0.51)	0.526	1.10 (0.82-1.48)
TG	56.99 (0.14)	54.98 (0.16)	0.362	0.83 (0.56-1.24)	54.98 (0.16)	47.98 (0.13)	0.528	1.26 (0.83-1.92)
*IFNGR1*								
AGTC	53.00 (0.13)	33.00 (0.10)	0.197	1.35 (0.85-2.15)	33 (0.10)	46 (0.13)	0.234	0.75 (0.47-1.21)
GATC	165.00 (0.40)	146.00 (0.42)	0.341	0.87 (0.65-1.16)	146 (0.42)	129.00 (0.35)	0.033	1.39 (1.03-1.89)
GGCT	101.63 (0.24)	92.00 (0.27)	0.394	0.87 (0.63-1.20)	92.00 (0.27)	107.00 (0.29)	0.538	0.90 (0.65-1.25)
GGTC	2.63 (0.01)	6.00 (0.02)	—	—	6.00 (0.02)	1 (0.01)	—	—
GGTT	95.37 (0.23)	67.00 (0.20)	0.295	1.21 (0.85-1.72)	67 (0.20)	83 (0.23)	0.345	0.84 (0.59-1.21)

CI: confidence interval; OR: odds ratio; PTB: pulmonary tuberculosis; LTBI: latent tuberculosis infection; HCS: healthy control subjects.

**Table 5:** Power of the study with different odds ratios (OR) in an allelic model.

SNPs	MAF		Power in PTB vs. LTBI			Power in LTBI vs. HC		
	LTBI	HC	OR = 2	OR = 3	OR = 4	OR = 2	OR = 3	OR = 4
*IFNG*								
rs1861494T>C	0.30	0.36	0.996	1	1	0.996	1	1
rs2069718A>G	0.17	0.13	0.997	1	1	0.937	1	1
*IFNGR1*								
rs1327475G>A	0.10	0.13	0.899	1	1	0.937	1	1
rs2234711G>A	0.41	0.34	0.997	1	1	0.995	1	1
rs3799488T>C	0.27	0.29	0.995	1	1	0.992	1	1
rs9376267C>T	0.46	0.43	0.997	1	1	0.996	1	1

SNP: single-nucleotide polymorphism; PTB: pulmonary tuberculosis; OR: odds ratio; LTBI: latent tuberculosis infection; HC: healthy controls; MAF: minor allele frequency.

## Data Availability

The datasets of the current study are available from the corresponding author on reasonable request.
